# Central obesity is selectively associated with cerebral gray matter atrophy in 15,634 subjects in the UK Biobank

**DOI:** 10.1038/s41366-021-00992-2

**Published:** 2022-02-10

**Authors:** Chris-Patrick Pflanz, Daniel J. Tozer, Eric L. Harshfield, Jonathan Tay, Sadaf Farooqi, Hugh S. Markus

**Affiliations:** 1 University of Cambridge Stroke Research Group, Neurology Unit, Department of Clinical Neurosciences, Cambridge Biomedical Campus, Cambridge, CB2 0QQ UK; 2 University of Cambridge Metabolic Research Laboratories and NIHR Cambridge Biomedical Research Centre, Welcome Trust-MRC Institute of Metabolic Science, Addenbrooke’s Hospital, Cambridge, CB2 0QQ UK

**Keywords:** Obesity, Obesity

## Abstract

**Background:**

Obesity is a risk factor for both cardiovascular disease and dementia, but the mechanisms underlying this association are not fully understood. We examined associations between obesity, including estimates of central obesity using different modalities, with brain gray matter (GM) volume in the UK Biobank, a large population-based cohort study.

**Methods:**

To determine relationships between obesity and the brain we used brain MRI, abdominal MRI, dual-energy X-ray absorptiometry (DXA), and bioelectric whole-body impedance. We determined whether obesity was associated with any change in brain gray matter (GM) and white matter (WM) volumes, and brain network efficiency derived from the structural connectome (wiring of the brain) as determined from diffusion-tensor MRI tractography. Using Waist-Hip Ratio (WHR), abdominal MRI and DXA we determined whether any associations were primarily with central rather than peripheral obesity, and whether associations were mediated by known cardiovascular risk factors. We analyzed brain MRI data from 15,634.

**Results:**

We found that central obesity, was associated with decreased GM volume (anthropometric data: *p* = 6.7 × 10^−16^, DXA: *p* = 8.3 × 10^−81^, abdominal MRI: *p* = 0.0006). Regional associations were found between central obesity and with specific GM subcortical nuclei (thalamus, caudate, pallidum, nucleus accumbens). In contrast, no associations were found with WM volume or structure, or brain network efficiency. The effects of central obesity on GM volume were not mediated by C-reactive protein or blood pressure, glucose, lipids.

**Conclusions:**

Central body-fat distribution rather than the overall body-fat percentage is associated with gray matter changes in people with obesity. Further work is required to identify the factors that mediate the association between central obesity and GM atrophy.

## Introduction

Obesity is defined as an excess of body fat that adversely effects health and is rising in prevalence globally. It is well recognized that obesity is associated with type-2 diabetes and cardiovascular disease [1]. Increasing evidence suggests effects on brain function with links reported between obesity and both cognitive function and dementia [2]. Effects on brain structure have been suggested to underlie these associations.

Obesity has been associated with global cerebral gray matter (GM) atrophy [3], but inconsistent associations with white matter (WM) volume have been published with reports of both decreases [4] and increases [5, 6]. More recent research used diffusion-tensor MRI tractography to reconstruct the connectome (characteristic wiring of the human brain at the mesoscale), and derived measures of brain network integrity that essentially indicate the robustness of the connectome against fault. These network metrics have been shown to correlate better with cognition than macrostructural WM volume, or WM hyperintensities (WMH) in both normal ageing [7] and disease states [8] and might be a more sensitive marker of white matter damage in obesity.

Assessing the effect of obesity on the brain is complicated by differing consequences of central or abdominal (high android-to-gynoid ratio which represents an increase in visceral fat around abdominal organs), and peripheral or subcutaneous (low android-to-gynoid ratio) obesity [9, 10]. The former has been particularly associated with metabolic syndrome [11], type-2 diabetes, myocardial infarction [12], and Alzheimer’s disease [13]. The effects of central obesity on the central nervous system (CNS) are less well understood.

As precise measurements of body fat and fat distribution are challenging to perform at scale, in clinical practice, anthropometric measures such as BMI (body mass index: weight in kg/height in m^2^) and waist-to-hip ratio (WHR) are used. However, abdominal MRI provides a much more reliable assessment of body-fat distribution, including the distinction between subcutaneous and visceral fat [14], although unless whole-body MRI is performed this does not allow quantification of total body fat. A further technique, dual-energy X-ray absorptiometry (DXA), can be used to obtain high-precision whole-body scans that provide accurate estimates of the body composition including the three major body components: fat mass, lean tissue mass, and bone mineral mass [15]. Whole-body bioelectrical impedance measures also provide indirect estimates of body fat.

To investigate associations between fat mass and fat distribution and the brain we combined the use of brain MRI, abdominal MRI, DXA, and bioelectric whole-body impedance. We determined whether [1] obesity/fat distribution was associated with any change in GM or WM volumes [2], with impairment of brain network efficiency derived from diffusion-tensor MRI and whether any associations were mediated by cardiovascular risk factors or blood biochemistry markers.

We also determined the relationship between markers of obesity with brain regions including the basal ganglia including caudate, putamen, pallidum and their major output nucleus the thalamus, as well as hippocampal volume as hippocampal atrophy has previously been reported in obesity [16].

## Methods

### Study participants

UK Biobank is a population-based cohort study comprising ~500,000 men and women aged 40–69 years, recruited across the United Kingdom (England, Scotland, and Wales) between 2006 and 2010 [17]. Following an initial assessment, a subset of participants returned for a neuroimaging visit that included brain 3.0T MRI, abdominal 1.5T MRI, and dual-energy X-ray absorptiometry (DXA) an average of 7.7 (SD = 1.4) years later. We used data from 15,634 subjects attending this imaging visit in this analysis.

To assess obesity, we used data on BMI and WHR available in all cases, as well as abdominal MRI (*N* = 15,634), DXA (*N* = 4,286), and bioelectric whole-body impedance data (*N* = 15,868). Availability of cognitive scores that were suitable for analysis varied from *N* = 15,631 for the visual memory and *N* = 7519 for the trial making task.

UK Biobank received ethical approval from the Research Ethics Committee (reference 11/NW/0382), and all participants provided written informed consent. The present analyses were conducted under UK Biobank application 36509.

### Measures of obesity

The following measures of body composition were used, all available as imaging-derived phenotypes in the UK Biobank dataset.

- BMI and WHR: UK Biobank has an anthropometry category that includes data on manually obtained body composition measure. We used BMI from the second assessment visit (to correspond to the imaging assessment) and calculated WHR as waist circumference divided by hip circumference.
- Whole-body bioelectrical impedance measures that were acquired using the Tanita BC418MA body composition analyzer (Tanita Corp., Tokyo, Japan), including segmental estimates of fat mass, fat-free mass.
- Abdominal MRI: We used imaging-derived indicators of abdominal composition, namely visceral adipose tissue, abdominal subcutaneous adipose tissue, total adipose tissue volume and total lean tissue volume, derived from abdominal 1.5T-MRI. All abdominal MRI measurements were performed using Siemens 1.5T MAGNETOM Aera (Siemens, Munich, Germany) using two pulse sequences to acquire the data: the first sequence consisted of a single breath-hold cardiac-gated T1-mapping Modified Look-Locker Inversion Recovery (MOLLI) sequence (typically 12 s), which acquires a series of seven images (8 mm slice thickness, in-plane pixel spacing 9.3 mm) each with a different T-weighting [18]. A single transverse slice located at the porta hepatis was chosen to represent the liver. Indices of body composition derived from the abdominal MRI data were supplied by AMRA (Advanced MR Analytics AB, AMRA, Sweden) according to described methods [14, 19].
- Dual X-ray absorptiometry (DXA): We used imaging-derived phenotypes of body composition, namely android-to-gynoid fat mass ratio, visceral adipose tissue mass, trunk-to-leg fat mass, trunk-to-leg lean mass, fat mass index, and lean body mass index, derived from DXA. DXA data were acquired using an iDXA instrument (GE-Lunar, Madison, Wisconsin) and measures of lean and fat mass were determined. The iDXA instrument was calibrated to a manufacturer’s phantom (GE-Lunar, Madison, Wisconsin) and underwent a daily QC procedure.

### Measures of brain structure

Brain MRI scans were acquired on a standard Siemens Skyra 3T (Siemens, Munich, Germany).

#### Brain volumes and white matter hyperintensities

We used the cerebral GM and WM volumes image-derived phenotypes in the UK Biobank dataset. These were derived from T1-weighted images and analyzed by an image-processing pipeline developed and run on behalf of UK Biobank [18]. We used volumes for specific brain regions: the caudate nucleus, putamen, pallidum, thalamus, hippocampus, that were derived from the structural T1-weighted images using FAST (FMRIB’s Automated Segmentation Tool) [20].

We analyzed the total volume of WM hyperintensities derived by UK Biobank using both T1 and T2-FLAIR images (*N* = 14662) and calculated with BIANCA [21].

#### Diffusion MRI and network construction

We derived network measures from the original diffusion MRI images. Diffusion-tensor MRI tractography was used to reconstruct the structural connectome (characteristic wiring of large tracts in the human brain) and brain network metrics were derived from the connectome that are indicative of how robust the brain network is against fault [22].

More specifically, diffusion-weighted images were corrected for eddy currents, head motion, outlier slices, and gradient distortion using FSL (FMRIB software library) [23]. Diffusion tensors were then fitted using the *b* = 1000 s/mm^2^ to get fractional anisotropy (FA) images for each subject. Each subjects’ FA image was non-linearly registered into standard space [18]. The FA image in diffusion space was then used as a seed for deterministic diffusion tractography carried out using MRtrix3 [24]. Termination criteria included: 20 mm < streamline length < 250 mm, turning angle > 45°, or voxel FA < 0.15 [25].

To construct networks we used the brain regions that are part of the Automated Anatomical Labeling (AAL) atlas [26] to generate adjacency matrices for each subject. These adjacency matrices fully describe the strength of connectivity node by node and represent the connectome at the level of the AAL atlas resolution. This atlas comprises 90 manually labeled cortical and subcortical areas (45 per hemisphere) in standard space, after having discarded cerebellar brain regions.

The tensorial field calculated from each participant’s FA image into standard space was inverted and then applied to the AAL using nearest-neighbor interpolation to register the AAL into diffusion space (where deterministic tractography was carried out). Two areas in the AAL were considered connected if joined by the endpoints of a reconstructed streamline, resulting in a non-zero edge in the adjacency matrix. Edges were weighted according to the number of streamlines connecting two regions, multiplied by the inverse average streamline length, as longer streamlines are seeded multiple times [27]. Edges weights <1 were zeroed to minimize noise-related false positives. This yielded a symmetric, undirected 90 × 90 adjacency matrix for each subject.

We used the adjacency matrix to compute global and local network efficiencies [28, 29], the most commonly used network metrics, using the brain graph package [30] and the igraph package available in R.

### Measures of cognitive performance

The cognitive tests used in our study were administered via touchscreen during the MRI visit. Visual memory was assessed using a pairs-matching test and scored as the total number of incorrect matches made. Reaction time was assessed using a timed symbol matching test similar to the card game ‘Snap’ and scored as the mean response time in milliseconds across all trials containing matching pairs. Prospective memory was assessed by giving participants an instruction they had to remember later in the assessment and scored as 1 if the participant remembered the instruction of their first try or 0 if not. Visual attention and task switching was assessed using a standard Trail Making Test.

### Blood biochemistry

Blood samples were collected at recruitment (for all 500,000 participants) and repeat assessment ~5 years later (for 20,000 participants), measuring a range of key biochemistry markers [31]. Blood glucose, high-density lipoprotein cholesterol, low-density lipoprotein cholesterol and glycated hemoglobin (HbA1c) were drawn from the blood biochemistry assessment at the first visit. The biomarkers were selected for analysis because they represent established risk factors associated with obesity and metabolic syndrome.

Within the UK Biobank study design, two blood pressure measurements were performed using automated or manual devices. We used the manual blood pressure measurement from the first assessment visit for the data analysis described below.

### Statistical data analysis

Statistical analyses were carried out using Python’s statistical, computing, machine learning packages SciPy [32] and Python’s stats package penguin [33]

To investigate the effects of obesity as determined from BMI and WHR measurements, and differentiate the effects of central and peripheral obesity, we stratified participants into 6 groups using previously reported cut-offs: [9] 1. Normal weight and no central obesity (males: 18.5 < BMI < 25, WHR < 0.9, females: 18.5 < BMI < 25, WHR < 0.8), 2. Normal weight and central obesity (males: 18.5 < BMI < 25, WHR > 0.9, females: 18.5 > BMI < 25, WHR > 0.8), 3. Overweight and no central obesity (males: 25 < BMI < 30, WHR < 0.9, females: 25 < BMI < 30, WHR < 0.9), 4. Overweight and central obesity (males: 25 < BMI < 30, WHR > 0.8. females: 25 < BMI < 30, WHR > 0.9), 5. General obesity and no central obesity (males: BMI > 30, WHR < 0.9, females: BMI > 30, WHR < 0.8), 6. Both general and central obesity (males: BMI > 30, WHR > 0.9, females: BMI > 30, WHR > 0.80. Subjects who were markedly underweight which may represent cachexia due to another cause (i.e., had a BMI < 18.5) were excluded.

We determined differences between groups for the brain measures: total brain volume (normalized for head size, from T1 images), cerebral GM volume (normalized for head size), WM volume (normalized for head size), log-transformed WM hyperintensities (normalized for head size), normalized network metrics, and cognitive scores. Analysis of covariance was used to test for group differences in neuroimaging outcomes across the stratified BMI/WHR groups using the following covariates sex, age, Townsend-Deprivation index (TDI), alcohol intake, current smoking, diabetes mellitus, systolic blood pressure, diastolic blood pressure and glycated hemoglobin (HbA1c). Partial correlations were used to test for associations between continuous indicators of obesity and neuroimaging outcomes after adjusting for the same set of covariates.

We also conducted a region-of-interest (ROI) analysis on regional brain volumes, derived from T1 images (see above). Due to previous data implicating basal ganglia circuits and the hippocampus in obesity we studied the following brain regions: caudate, putamen, pallidum, as well as the thalamus, amygdala, nucleus accumbens, and hippocampus. Basal ganglia dysfunction [34] and alterations in amygdala and thalamus [35] and hippocampal volumes [16] have been reported in obesity. The nucleus accumbens plays a role in food addiction [36] and is a target for deep brain stimulation in severe obesity [37].

In a second analysis, anthropometric data (BMI and WHR), impedance measures of body fat and lean body mass, and imaging (abdominal MRI and DXA scans) derived measures of general and central obesity were used as continuous predictors to investigate their effect on the normalized cerebral GM volume using the same covariates as mentioned above. We then determined whether indicators from blood biochemistry or blood pressure mediated the effect of obesity on the normalized GM volume, by running a mediation analysis using IBM SPSS Amos for Structural Equation Modeling [38].

## Results

### Sample size

Subjects were included in the data analysis if imaging outcomes and data of the following co-variates were available: sex, age, TDI, alcohol intake, current smoking, diabetes mellitus, systolic blood pressure, diastolic blood pressure and HbA1c.

Data were available for the following number of subjects: Brain volumes *N* = 15,634; multimodal WMH (based on FLAIR and T1 images) *N* = 14,662; DTI and network analysis: 14,368. BMI and WHR were available for all subjects who underwent brain imaging. Data on assessment of obesity was available for bioelectric impedance analysis *N* = 15,437; abdominal MRI *N* = 5155; DXA *N* = 4212.

### BMI, WHR and brain measures

**Fig. 1:**
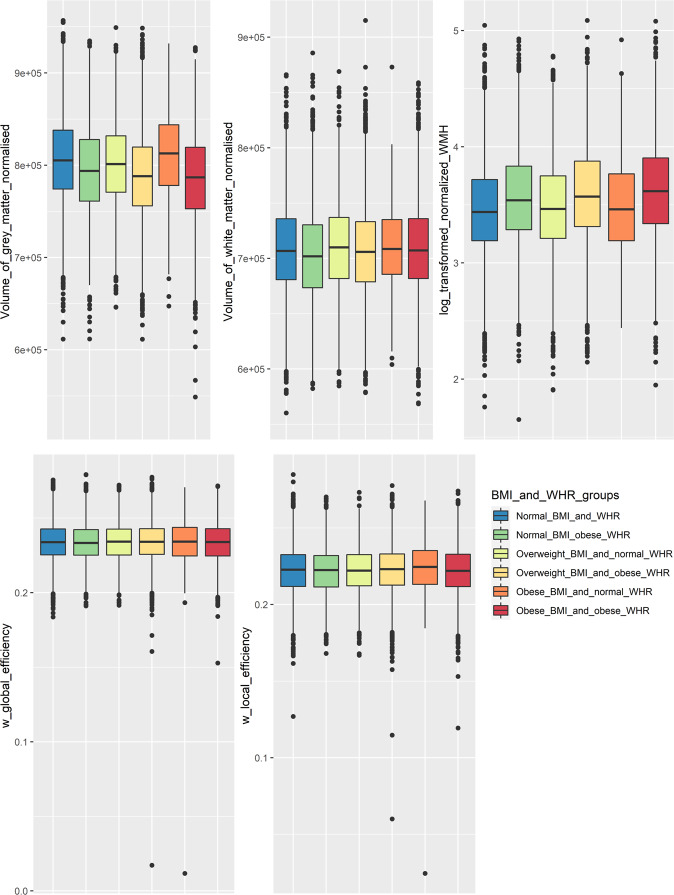
Association between BMI and WHR with GM and WM volumes, WMH and brain network measures (global and local efficiency). Significant differences across the stratified BMI and WHR groups are present for GM volume and WMH but not for WM volumes or network measures. (Error bars show standard deviations, boxes correspond to the interquartile range.).

Unless otherwise stated results are adjusted for the covariates (see methods section). There was a significant progressive reduction in cerebral GM volume as WHR increased, with WHR rather than BMI being the primary driver of this association, with GM volume being lowest in people with overweight and central obesity (*p* = 6.7 × 10^−16^, η_p_^2^ = 0.004, see Fig. 1). The association of combined BMI/WHR group and GM volume appeared to be related to a loss of normalized brain volume (*p* = 2.2 × 10^−16^, η_p_^2^ = 0.011). In contrast, there was no association with WM volume (*p* = 0.135, η_p_^2^ = 0.001). Furthermore, there was no overall effect of combined BMI/WHR group with any brain network measure: weighted global efficiency (*p* = 0.594, η_p_^2^ = 0.001, see Fig. 1), weighted local efficiency (*p* = 0.607, η_p_^2^ = 0.001). There was a weak association with WM hyperintensities (*p* = 2.4 × 10^−32^, η_p_^2^ = 0.011).

Next associations between obesity groups were performed for individual cerebral GM regions. After adjustment for global GM volume, significant associations remained with the following regions: bilateral thalamus, bilateral caudate, bilateral pallidum, bilateral nucleus accumbens, after accounting for Bonferroni-corrected *p* value of 0.004. Associations with bilateral putamen, bilateral amygdala, and bilateral hippocampus were not significant after Bonferroni correction (see Table 1).

**Table 1:** Analysis of covariance looking at the overall effect of the combined BMI/WHR group on the volumes of subcortical regions-of-interest before and after adjustment for total GM volume.

Region of interest	Hemisphere	Results adjusted for covariates				Results adjusted for GM atrophy and covariates			
		**F (5, 15,619)**	***p*** **value**	**η**_**p**_^**2**^		**F (5, 15,619)**	***p*** **value**	**η**_**p**_^**2**^	
Thalamus	Left	**6.618**	**3.658** **×** **10**^**−6**^	**0.002**	*	**4.461**	**4.606** **×** **10**^**−4**^	**0.001**	*
Thalamus	Right	**7.056**	**1.345** **×** **10**^**−6**^	**0.002**	*	**4.921**	**1.672** **×** **10**^**−4**^	**0.001**	*
Caudate	Left	**5.98**	**1.559** **×** **10**^**−5**^	**0.001**	*	**5.235**	**8.306** **×** **10**^**−5**^	**0.001**	*
Caudate	Right	**6.943**	**1.741** **×** **10**^**−6**^	**0.002**	*	**6.164**	**1.027** **×** **10**^**−5**^	**0.002**	*
Putamen	Left	2.752	0.017	0.001		1.695	0.131	5.43 × 10^−4^	
Putamen	Right	2.668	0.02	8.542 × 10^−4^		1.478	0.193	0.0004	
Pallidum	Left	**7.238**	**8.838** **×** **10**^**−7**^	**2.314** **×** **10**^**−3**^	*	**7.24**	**8.798** **×** **10**^**−7**^	**2.315** **×** **10**^**−4**^	*
Pallidum	Right	**10.244**	**8.074** **×** **10**^**−10**^	**0.003**	*	**9.356**	**6.489** **×** **10**^**−9**^	**0.002**	*
Amygdala	Left	2.751	0.017	8.81 × 10^−4^		2.733	0.018	0.001	
Amygdala	Right	**3.804**	**0.001**	**0.001**	*	**3.626**	**0.0028**	**0.001**	*
Nucleus accumbens	Left	**8.4318**	**5.606** **×** **10**^**−8**^	**0.002**	*	**4.886**	**1.805** **×** **10**^**−4**^	**0.001**	*
Nucleus accumbens	Right	**9.884**	**1.882** **×** **10**^**−9**^	**0.003**	*	**5.676**	**3.098** **×** **10**^**−5**^	**0.001**	*
Hippocampus	Left	0.927	0.461	2 × 10^−3^		0.586	0.071	1.88	
Hippocampus	Right	2.546	0.026	0.001		2.563	0.025	0.001	

*P* values in bold, and marked with *, are those significant after Bonferroni correction at *p* value 0.004.

### Association with indicators of body-fat mass

#### Bioelectrical impedance

**Fig. 2:**
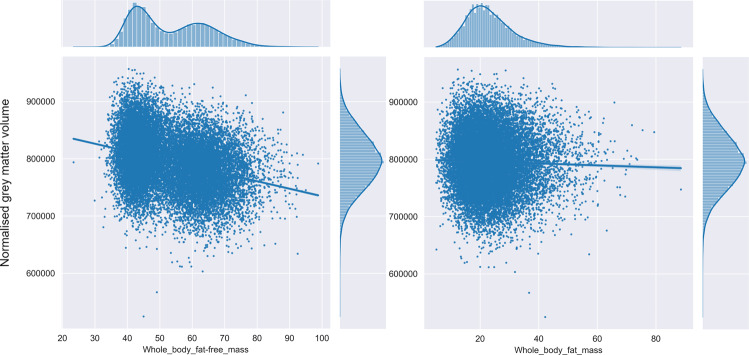
Scatter plots showing the significant negative effect of whole-body fat-free mass and whole-body fat mass on the normalized cerebral GM volume. Pearson r in these figures corresponds to the overall effect size of whole-body fat-free/fat mass on cerebral GM volume before correction for the covariates.

Higher body-fat mass was associated with lower GM volume (see Fig. 2), as well as lower total brain volume, but not with WM volume (see Table 2).

**Table 2:** Partial correlations between whole-body fat mass and whole-body fat-free mass from bioelectrical impedance analysis and neuroimaging outcomes adjusted for covariates.

Bioelectical impedance	Neuroimaging outcome	*r*	CI 95%	Adjusted variance explained	*p* value	
Whole-body fat mass	Brain volume	**−0.07**	**[−0.09, −0.06]**	**0.005**	**1.772** **×** **10**^**−19**^	*
	GM volume	**−0.11**	**[−0.13, −0.1]**	**0.012**	**2.925** **×** **10**^**−47**^	*
	WM volume	−0	[−0.02, 0.01]	−0.0001	0.533	
	Global Efficiency	0.001	[−0.01, 0.02]	−0.0001	0.823	
	Local Efficiency	0.001	[−0.02, 0.02]	−0.0001	0.887	
Whole-body-fat free mass	Brain volume	**−0.12**	**[−0.14, −0.11]**	**0.014**	**1.439** **×** **10**^**−53**^	*
	GM volume	**−0.17**	**[−0.18, −0.15]**	**0.028**	**5.772** **×** **10**^**−101**^	*
	WM volume	**−0.03**	**[−0.05, −0.02]**	**0.0009**	**0.000037**	*
	Global Efficiency	0.004	[−0.01, 0.02]	−0.0001	0.588	
	Local Efficiency	0.003	[−0.01, 0.02]	−0.0001	0.688	

Significant results, at the *p* = 0.05 level, are shown in bold and with *.

#### Abdominal MRI

**Fig. 3:**
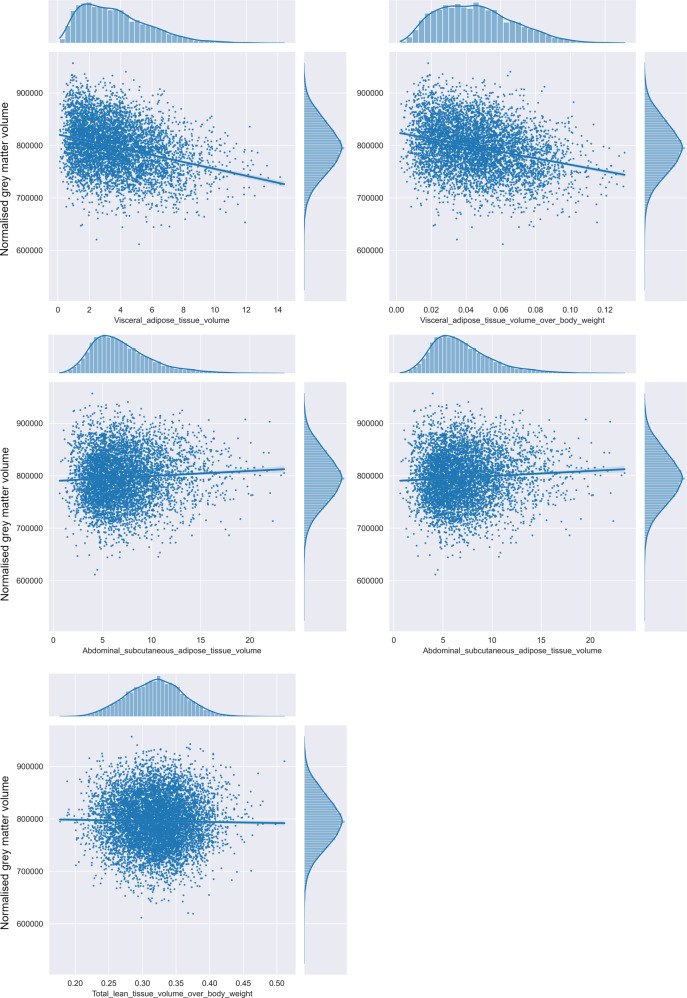
Associations between indicators of body composition from abdominal MRI and normalized GM volume. Visceral adipose tissue volume (normalized by body weight), abdominal subcutaneous adipose tissue volume, total adipose tissue volume and total lean tissue volume (normalized by body weight), derived from abdominal MRI, as predictors of normalized cerebral GM volume. Pearson *r* in these figures corresponds to the overall effect size of the association before correction for the covariates.

Higher total adipose tissue volume was associated with lower GM volume as well as lower total brain volume (see Table 3 and Fig. 3). By contrast, no association was found between total adipose tissue volume and WM volume (see Table 3).

**Table 3:** Partial correlations between indicators of body composition derived from abdominal MRI and neuroimaging outcomes adjusted for covariates.

Abdominal MRI	Brain MRI	*r*	CI 95%	Adjusted variance explained	*p* value	Significant
Visceral adipose tissue volume	Brain volume	**−0.072**	**[−0.1, −0.05]**	**0.00486**	**1.928** **×** **10**^**−07**^	*
	GM volume	**−0.096**	**[−0.12, −0.07]**	**0.00888**	**4.279** **×** **10**^**−12**^	*
	WM volume	0.023	[0.05, 0.0]	0.00018	0.085	
	Global efficiency	−0.011	[−0.02, 0.04]	0.00031	0.441	
	Local efficiency	−0.018	[−0.01, 0.05]	0.0001	0.217	
Abdominal subcutaneous adipose tissue volume	Brain volume	**−0.067**	**[−0.09, −0.04]**	**0.00419**	**0.000001**	*
	GM volume	**−0.094**	**[−0.12, −0.07]**	**0.0085**	**1.178** **×** **10**^**−11**^	*
	WM volume	−0.018	[−0.05, 0.01]	0.00004	0.184	
	Global efficiency	0.015	[−0.01, 0.05]	0.00019	0.289	
	Local efficiency	0.01	[−0.02, 0.04]	0.00034	0.498	
Total adipose tissue volume	Brain volume	**−0.112**	**[−0.13, −0.09]**	**0.012**	**2.679** **×** **10**^**−21**^	*
	GM volume	**−0.08**	**[−0.1, −0.06]**	**0.00614**	**1.157** **×** **10**^**−11**^	*
	WM volume	−0.021	[−0.04, 0.0]	0.00016	0.075	
	Global efficiency	0.006	[−0.02, 0.03]	0.00028	0.649	
	Local efficiency	3E-04	[−0.02, 0.02]	0.0003	0.979	
Lean tissue volume (normalized by body weight)	Brain volume	**0.048**	**[0.03, 0.07]**	**0.002**	**0.00004**	*
	GM volume	**0.046**	**[0.02, 0.07]**	**0.001878**	**0.000093**	*
	WM volume	**0.032**	**[0.01, 0.06]**	**0.000762**	**0.0065**	*
	Global efficiency	0.005	[−0.02, 0.03]	0.000283	0.649	
	Local efficiency	3E−04	[−0.02, 0.02]	0.000316	0.979	

Significant results, at the *p* = 0.05 level, are shown in bold and with *.

#### DXA

Fat-mass index (FMI) showed a significant negative effect on GM volume as well as total brain volume and but not on WM volume after adjusting for the covariates (see Supplementary Fig. 2 and Supplementary Table 1).

No association was found between network metrics and any indicator of body-fat mass irrespective of imaging modality

### Association with indicators of lean body mass

#### Bioelectrical impedance

Higher whole-body-fat free mass was associated with lower GM volume, as well as lower total brain volume (see Fig. 2 and Table 2). Similarly, a negative association was found between whole-body-fat free mass and WM volume (see Table 2).

#### Abdominal MRI

Lean tissue volume (normalized by body weight) was not associated with any difference in GM volume after adjusting for the covariates. Weak correlations were found between total lean tissue volume (normalized by body weight) and normalized WM volume as well as normalized total brain volume (see Table 3 and Fig. 3).

#### DXA

Lean body-mass index (LBMI) showed a significant negative effect on GM volume as well as total brain volume and but not on WM volume after adjusting for the covariates (see Supplementary Fig. 2 and Supplementary Table 1).

No association was found between network metrics and any indicator of lean body mass irrespective of imaging modality.

### Association with indicators of fat distribution

#### Abdominal MRI

Higher visceral adipose tissue volume was associated with lower total brain volume. As for WHR, this was explained by a significant negative correlation with GM volume after adjusting for covariates, before and after normalization by each subject’s body weight (*p* = 0.001, Fig. 3), but there was no association with WM volume (Table 3 and Fig. 3).

Similarly, abdominal subcutaneous adipose tissue volume was negatively associated with GM volume and total brain volume (Table 3 and Fig. 3). However, no significant correlation was found between abdominal subcutaneous adipose tissue volume and WM volume.

#### DXA

Higher visceral adipose tissue mass was associated with lower GM volume and total brain volume, but not with WM volume or network metrics (Supplementary Table 1).

There was a significant negative correlation between android-to-gynoid fat ratio and GM volume before adjusting for the covariates (*r* = −0.23, *p* = 1.3 × 10^−57^), but this effect was not significant after adjusting for covariates (*r* = 0.022, 95% CI = [−0.01, 0.05]). Associations between android-to-gynoid fat ratio and WM volume were significant, but small in magnitude.

Trunk-to-leg fat mass ratio showed a significant negative correlation with GM volume before adjusting for the covariates (*r* = −0.27, *p* = 8.3 × 10^−81^). However, the trunk-to-leg fat mass ratio did not show a significant association with GM volume, total brain volume, or WM volume after adjusting for the covariates (Supplementary Table 1).

No association was found between network metrics and any indicator of fat distribution irrespective of imaging modality.

### Association with cognitive scores

Analysis of co-variance did not reveal any significant effects of combined BMI/WHR group on the following cognitive scores: log-transformed number of incorrect matches in the visual memory task (*p* = 0.313), log-transformed difference between Trail making test – Part B and Trail making test – Part A (*p* = 0.388), the prospective memory task score (*p* = 0.756), and the number of correctly made symbol digit matches (*p* = 0.891). The effect of combined BMI/WHR on the log-transformed, average time to correctly identify matches was not significant after correcting for multiple comparisons (uncorrected *p* = 0.001).

### Mediation analysis using structural equation modeling

The effect of WHR on GM volume was not mediated by blood glucose, glycated hemoglobin (HbA1c), HDL cholesterol, systolic or diastolic blood pressure (all *p* > 0.05, Supplementary Figs. 3 and 4). Similarly, the effects of visceral adipose tissue volume from abdominal MRI and visceral adipose tissue mass from dual X-ray absorptiometry were not mediated by any of the aforementioned mediators (Supplementary Fig. 5).

#### Interactions between indicators of obesity and sex

Finally, we investigated interaction effects between indicators of obesity and sex when predicting GM, WM, WMH and total brain volumes (Supplementary Table 2). We found significant interaction effects between sex and body fat mass, body fat-free mass (impedance), abdominal subcutaneous adipose tissue volume (abdominal MRI) as well as adipose tissue volume (abdominal MRI). By contrast, no interaction with gender was found for android-to-gynoid fat mass ratio, trunk-to-leg fat mass ratio, or trunk-to-leg lean mass ratio.

## Discussion

In this large population-based study, central obesity was associated with lower GM volume, but not with WM volume or brain network integrity. We assessed central obesity using a variety of techniques including WHR, visceral adipose tissue from abdominal MRI, DXA, and bioelectrical impedance. Consistent results, with a selective association with GM volume, were found when central obesity was assessed by these diverse techniques. Furthermore, indicators of central obesity (e.g., WHR) were more informative than indicators of general obesity (e.g., BMI) in predicting brain volumes and being overweight alone, as measured using BMI, did not seem to have a detrimental effect on the brain.

Our findings add to the existing knowledge in a number of ways. Firstly, it is in a larger sample size than previous studies, including over 15,000 individuals. Secondly, we used multiple complementary methods for assessing central obesity (BMI/WHR, abdominal MRI, dual-energy X-ray absorptiometry (DXA), and bioelectric whole-body impedance) which increases the robustness of the findings. Thirdly, we showed the associations with central obesity and brain structure were specific for gray matter, and was not present with white matter or with brain network connectivity; associations with network connectivity have not previously been assessed to our knowledge.

Increasing evidence suggests that obesity affects the CNS, and cognitive function including attention, executive function, decision making, and verbal learning [39]. Meta-analyses have shown strong associations between obesity, Alzheimer’s disease, and other dementias, with obesity in midlife predicting future dementia risk [2, 40]. Postmortem studies have shown that elderly individuals with morbid obesity have increased beta-amyloid and tau protein in the hippocampus and decreased hippocampal volume [41]. However, how obesity links to impaired cognitive function and dementia remains uncertain. Previous studies, many in relatively small sample sizes, have shown associations between obesity and GM volume, but inconsistent associations with WM volume and WM ultrastructural function determined on DTI [4–6]

However, many of these have merely measured BMI as an estimate of obesity and have not looked at the fat distribution within the body. In central obesity, there is increased fat in the abdomen and internal organs that causes low-grade inflammation [39]. Central obesity has been associated with metabolic syndrome that includes dyslipidemia, decreased insulin sensitivity, hyperinsulinemia, hypoglycemia, and hypertension [12]. Our results suggest that it is central obesity rather than peripheral obesity that is associated with CNS damage.

While previous smaller studies have associated obesity with GM loss, there have been conflicting associations reported with WM volume and structure [4–6]. Since obesity has been associated with cognitive deficits including executive function [42], which itself depends on complex brain networks depending on WM integrity and connectivity, we hypothesized that brain network analysis may be affected in obesity. However, despite examining associations in almost 20,000 individuals we found no evidence of any alteration in WM volume or ultrastructure as measured by DTI and brain network efficiency. This suggests that CNS damage associated with central obesity is primarily focused on GM. This most likely represents neuronal loss rather than degeneration of tracts, because the cell bodies of neurons are found in the gray matter of the brain. Associations we have detected with GM volume might represent changes relatively early in the course of detrimental effects of central obesity on the brain, and as individuals age, more widespread changes may be identified with secondary axonal degeneration and WM changes. Longitudinal studies are required to determine if this is indeed the case.

While central obesity, perhaps acting via the metabolic syndrome, may result in brain atrophy, it is also possible that preexisting alterations in brain structure and function play a causal role in obesity itself. Several neural circuits have been described which are involved in energy balance and affect appetite and thermogenesis. Unconscious reward circuits involve the striatum, amygdala, hippocampus, substantia nigra, hypothalamus and brainstem which are all part of the dopaminergic mesocortical limbic circuit [39]. To determine whether there were specific regional associations with obesity we examined associations with specific subcortical GM regions and the hippocampus. We controlled for total GM volume to ensure that associations were region-specific. The analysis identified specific associations with thalami, the caudate nuclei, pallidum, and nuclei accumbens (all bilaterally). However, after controlling for multiple comparisons, no association was found with the putamen, amygdala, or hippocampus. The caudate nuclei, pallidum, and nuclei accumbens are known to play a key role in energy balance that affect appetite, thermogenesis as well as inhibitory control [39]. More specifically, the nucleus accumbens is involved in components of reward-motivated behaviors [43] as well as food addictions [36]. The caudate nucleus and pallidum play a role in the inhibitory control of eating [44] and contribute to physical inactivity in obesity [34]. Both the striatum and the nucleus accumbens (ventral striatum) are part of the dopaminergic mesocortical limbic circuit that plays a crucial role in unconscious reward [39] and has been suggested to be a common neurobiological circuit between food addiction and drug abuse [36].

We explored potential pathways by which central obesity might result in GM atrophy using mediation analysis. Central obesity has been associated with a low-grade inflammatory state. However, the association between central obesity and GM volume was not altered when C-reactive protein, a marker of systemic inflammation, was controlled for. It has also been hypothesized that the metabolic syndrome may mediate end-organ damage [45], but the associations of WHR, or visceral adipose tissue volume, determined either on MRI or DEXA, was not mediated by blood glucose, HbA1c, HDL cholesterol, systolic or diastolic blood pressure. This does not support a direct mediation by individual features of the metabolic syndrome. Further studies are required to determine which factors associated with central obesity mediate the association with GM atrophy.

Central obesity might lead to neuronal loss or shrinkage of neurons in the cerebral GM that is then not seen in the WM in the first place. Previous research on obesity in UK Biobank found that higher total body fat was associated with lower subcortical GM volumes, including the thalamus, caudate nucleus, putamen, globus pallidus, hippocampus, and nucleus accumbens [46]. These findings are similar to our results when looking at the negative effect of WHR on the whole brain and subcortical GM volume. A further UK Biobank paper showed that the combination of being overweight according to BMI criteria and central obesity as indicated by WHR was associated with lower GM volume, whereas no association between obesity and WM volume were found [9].

Our study has several strengths. This includes a large sample size and the population-based sampling framework. We estimated central obesity using multiple different techniques and showed consistency of findings when central obesity was determined by these different methods. This is important because each method has its limitations. DXA can be inaccurate in people with obesity. Impedance measures also depend on hydration levels. The body MRI data we were able to use only covered the abdomen rather than whole-body MRI and therefore an accurate estimate of whole-body fat could not be obtained. A further strength was that multiple methods were used to assess brain integrity, including not only conventional measures of GM and WM volume, but also DTI measures of network efficiency. The consistency of findings across WM measures adds robustness to the finding that WM structure does not appear to be altered in obesity.

Our study also has limitations. Its cross-sectional design means that we can identify associations but not causality. Although UK Biobank is a population-based study there is selection bias toward healthy volunteers. Lastly, although highly significant associations were identified, due to the very large sample size some of the effect sizes were small.

In conclusion, our results suggest that being overweight itself has only a limited effect on brain volume, but central obesity is associated with significant reductions in both global GM volume, and also with volume of specific subcortical GM nuclei.

## Supplementary information

Supplemental Material
