# Supplementary material for: Central obesity is selectively associated with cerebral gray matter atrophy in 15,634 subjects in the UK Biobank
Source: Int J Obes (Lond). 2022 Feb 10;46(5):1059–67. doi: 10.1038/s41366-021-00992-2 (PMC9050590; doi:10.1038/s41366-021-00992-2)
Supplement: Supplementary file 1 — Supplemental Material [file 41366_2021_992_MOESM1_ESM.docx]

**Supplementary Information**

**
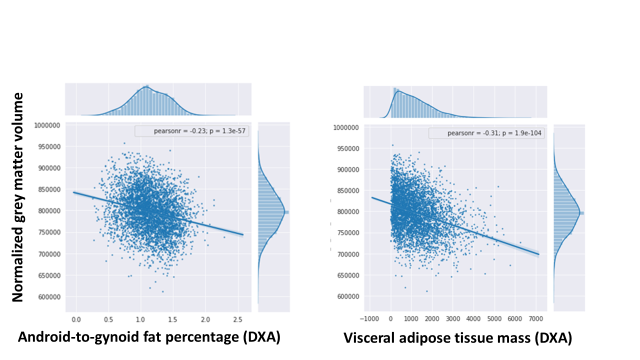
**

**
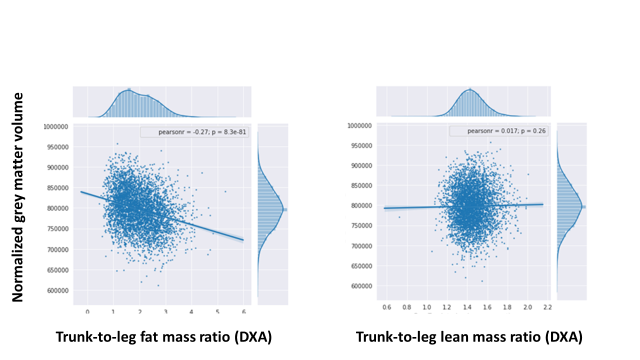

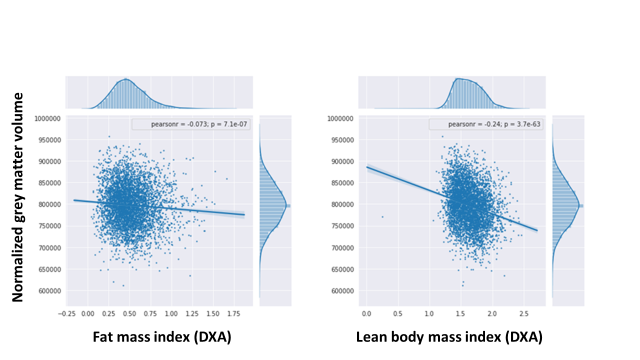
**

**Supplementary Figure 1.** Association between estimates of body composition form dual X-ray absorptiometry and normalized cerebral GM volume from the T1-weighted image. These include the android-to-gynoid fat percentage, visceral adipose tissue, trunk-to-leg fat mass, trunk-to-leg lean mass, fat mass index (FMI) and lean body mass index (LBMI). Pearson r in these figures corresponds to the overall effect size of the association before correction for the covariates.


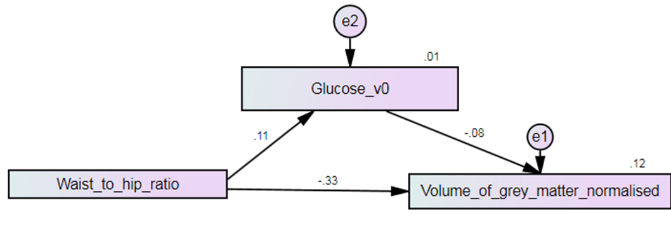

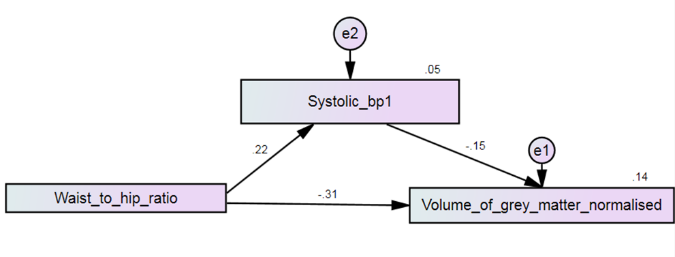


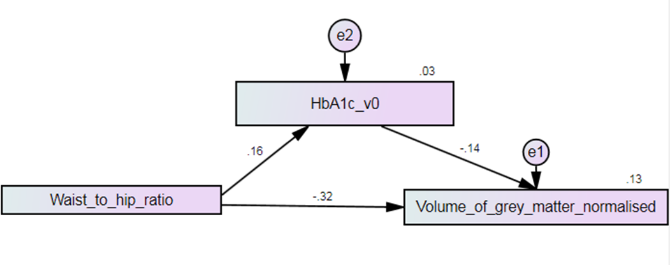

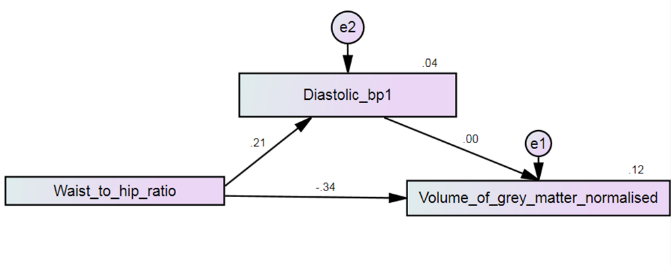


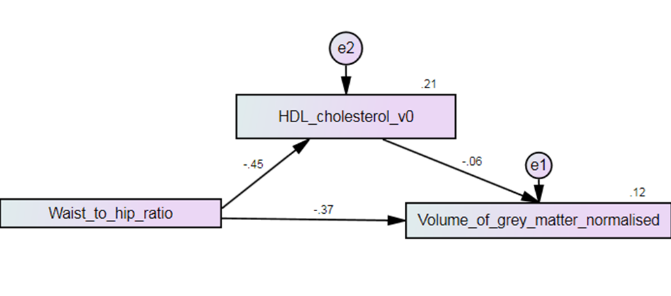

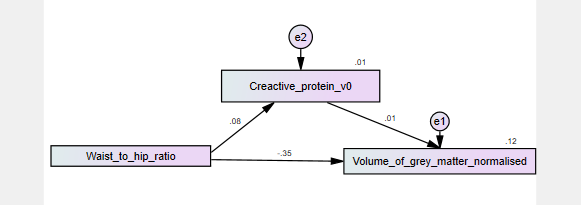


**Supplementary Figure 2.** Mediation analysis investigating if the effect of waist-to-hip ratio on the normalized volume of grey matter is mediated by glucose, HbA1c, HDL cholesterol, systolic blood pressure, diastolic blood pressure, or polygenetic risk scores for metabolic syndrome, C-reactive protein or central obesity (waist-to-hip ratio). Numbers on the arrows are standardized effect sizes, numbers next to the boxes indicate the variance explained.


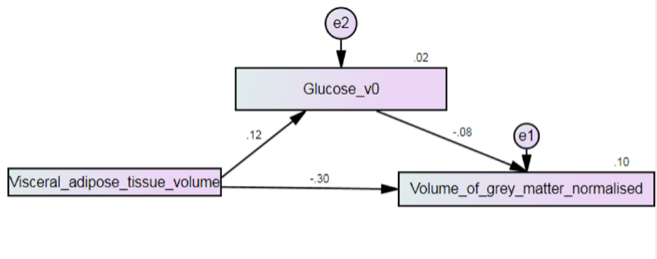

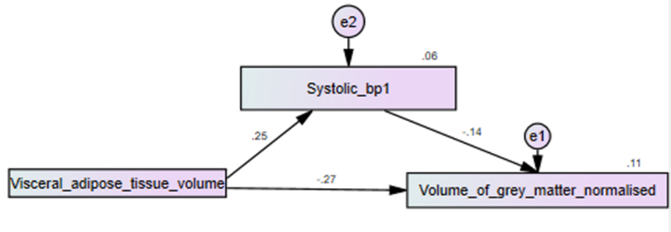


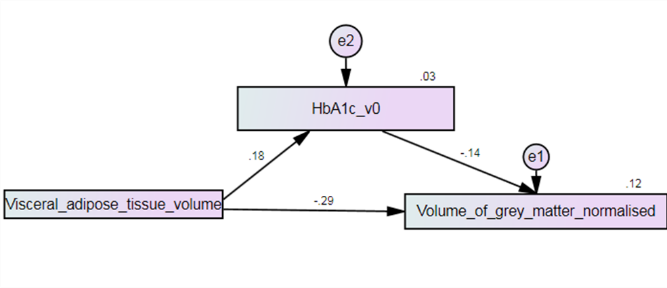

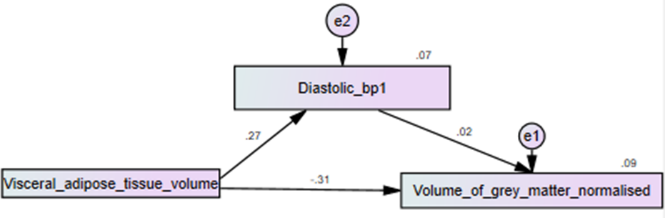


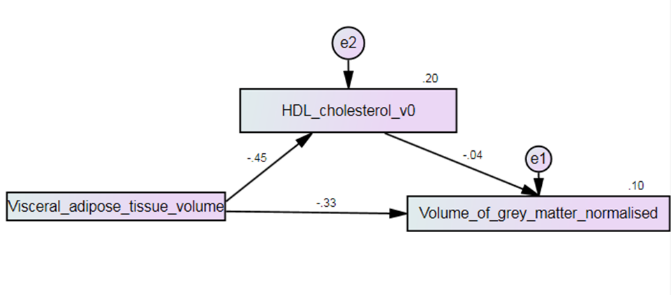

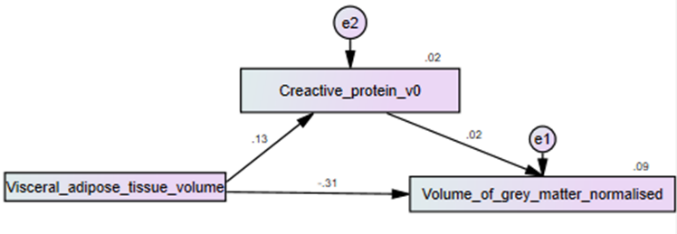


**Supplementary Figure 3.** Mediation analysis investigating if the effect of visceral adipose tissue volume (from abdominal MRI) on the normalized volume of grey matter is mediated by glucose, HbA1c, HDL cholesterol, systolic blood pressure, diastolic blood pressure, or polygenetic risk scores for metabolic syndrome, C-reactive protein or central obesity (waist-to-hip ratio). Numbers on the arrows are standardized effect sizes, numbers next to the boxes indicate the variance explained.

**
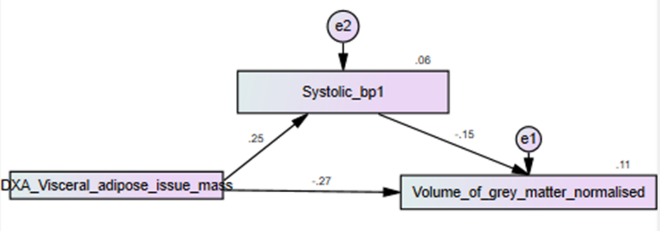

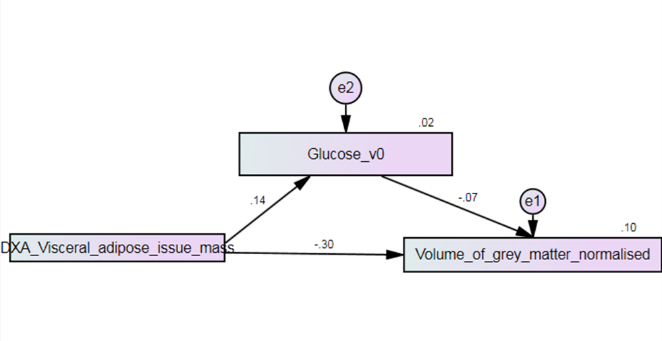

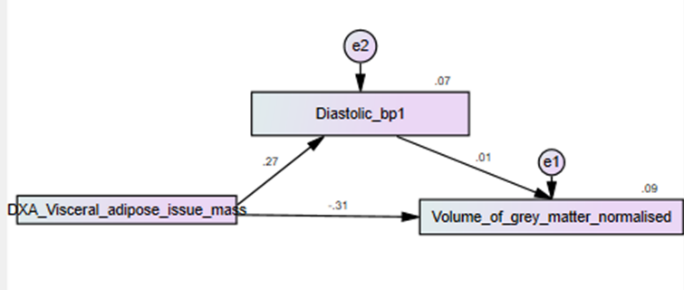
**

**
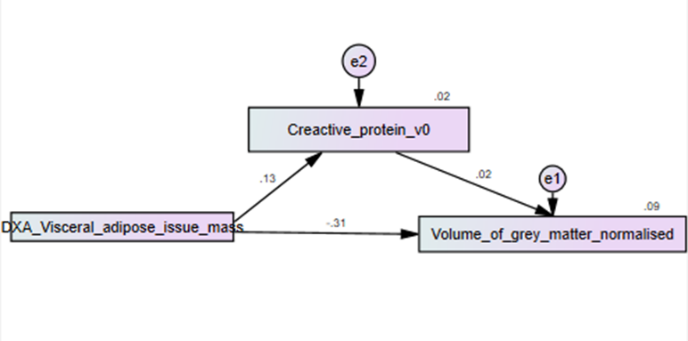

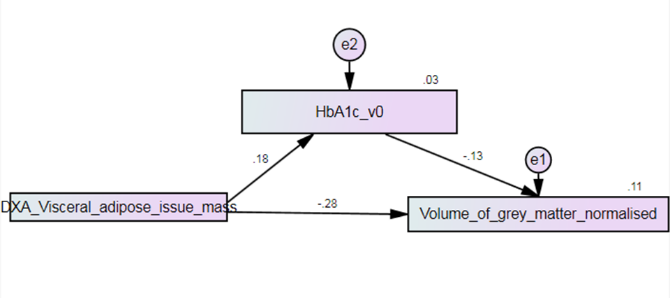
**

**
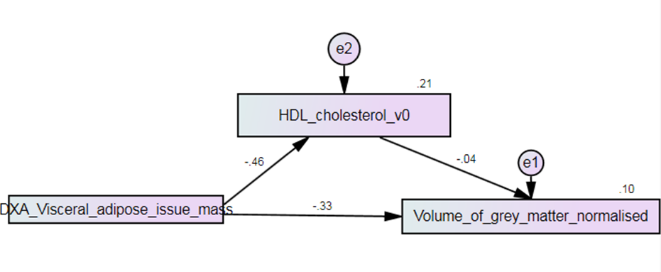

Supplementary Figure 4**. Mediation analysis investigating if the effect of visceral adipose tissue volume (from abdominal MRI) on the normalized volume of grey matter is mediated by glucose, HbA1c, HDL cholesterol, systolic blood pressure, diastolic blood pressure, or polygenetic risk scores for metabolic syndrome, C-reactive protein or central obesity (waist-to-hip ratio). Numbers on the arrows are standardized effect sizes, numbers next to the boxes indicate the variance explained.

|  |  | r | CI 95% | Adjusted variance explained | p-value |
| --- | --- | --- | --- | --- | --- |
| Android-to-gynoid fat ratio | **Brain** | **0.039483** | **[0.01, 0.07]** | **0.001093** | **0.009735** |
|  | GM | 0.022034 | [-0.01, 0.05] | 0.000019 | 0.149224 |
|  | **WM** | **0.040481** | **[0.01, 0.07]** | **0.001173** | **0.008037** |
|  | **WMH** | **0.064298** | **[0.03, 0.1]** | **0.00359** | **0.000099** |
|  | Eglob | 0.003626 | [-0.03, 0.04] | -0.000543 | 0.827826 |
|  | Eloc | -0.0044 | [-0.04, 0.03 | -0.000536 | 0.791827 |
| Visceral adipose tissue  mass | **Brain** | **-0.080866** | **[-0.11, -0.05]** | **0.00607** | **1.362866×10^-07^** |
|  | **GM** | **-0.121552** | **[-0.15, -0.09]** | **0.014309** | **2.050297×10^-15^** |
|  | WM | -0.014284 | [-0.04, 0.02] | -0.000268 | 0.352671 |
|  | **WMH** | **0.101866** | **[0.07, 0.13]** | **0.009829** | **8.241557×10^-10^** |
|  | Eglob | -0.001341 | [-0.03, 0.03] | -0.000561 | 0.936267 |
|  | Eloc | 0.00128 | [-0.03, 0.03] | -0.000561 | 0.93914 |
| Trunk-to-leg lean mass | Brain | 0.021972 | [-0.01, 0.05] | 0.000016 | 0.150371 |
|  | GM | 0.038772 | [0.01, 0.07] | 0.001037 | 0.011132 |
|  | WM | -0.001328 | [-0.03, 0.03] | -0.000465 | 0.930732 |
|  | WMH | -0.005261 | [-0.04, 0.03] | -0.000519 | 0.750266 |
|  | Eglob | -0.000072 | [-0.03, 0.03] | -0.000556 | 0.996542 |
|  | Eloc | 0.018881 | [-0.01, 0.05] | -0.000199 | 0.257319 |
| Trunk-to-leg fat mass ratio | Brain | 0.015648 | [-0.01, 0.05] | -0.000222 | 0.305748 |
|  | GM | -0.004211 | [-0.03, 0.03] | 0.000449 | 0.782855 |
|  | WM | 0.027689 | [-0.0, 0.06] | 0.0003 | 0.069896 |
|  | **WMH** | **0.084302** | **[0.05, 0.12]** | **0.006564** | **3.229847×10^-07^** |
|  | Eglob | 0.006417 | [-0.03, 0.04] | -0.000515 | 0.700279 |
|  | Eloc | 0.007245 | [-0.03, 0.04] | -0.000503 | 0.663836 |
| Fat mass  index | **Brain** | **-0.062884** | **[-0.09, -0.03]** | **0.003489** | **0.000038** |
|  | **GM** | **-0.0991** | **[-0.13, -0.07]** | **-0.009358** | **8.049703×10^-11^** |
|  | WM | -0.006885 | [-0.04, 0.02] | -0.00042 | 0.652375 |
|  | **WMH** | **0.097635** | **[0.07, 0.13]** | **0.008991** | **3.226189×10^-09^** |
|  | Eglob | 0.000193 | [-0.03, 0.03] | -0.000556 | 0.990788 |
|  | Eloc | -0.006616 | [-0.04, 0.03] | -0.000512 | 0.691529 |
| Lean  body  mass index | **Brain** | **-0.064625** | **[-0.09, -0.03]** | **0.003711** | **0.000023** |
|  | **GM** | **-0.10506** | **[-0.13, -0.08]** | **0.010575** | **5.474485×10^-12^** |
|  | WM | -0.004183 | [-0.03, 0.03] | -0.00045 | 0.784348 |
|  | **WMH** | **0.0465** | **[0.01, 0.08]** | **0.001617** | **0.004892** |
|  | Eglob | -0.008219 | [-0.04, 0.02] | -0.000489 | 0.622076 |
|  | Eloc | 0.003615 | [-0.03, 0.04] | -0.000543 | 0.828365 |

**Supplementary Table 1.** Partial correlations between indicators of body composition derived from dual x-ray absorptiometry and neuroimaging outcomes adjusted for covariates.

| **Normalized**  **Volume of** | **Interaction effect** | **Effect size**  **(t-value)** | **Asymptotic**  **p-value** |
| --- | --- | --- | --- |
| **GM** | **Interaction effect between whole**  **body fat mass (impedance)**  **and sex** | **-8.0996** | **< 0.0001** |
| **WM** |  | **-2.6888** | **0.0072** |
| **WMH** |  | **6.9690** | **< 0.0001** |
| **Brain** |  | **-6.6303** | **< 0.0001** |
| GM | Interaction effect between whole_  Body fat-free mass (impedance)  and sex | -1.9099 | 0.0562 |
| **WM** |  | **-3.2740** | **0.0011** |
| **WMH** |  | **4.4336** | **< 0.0001** |
| **Brain** |  | **-3.0719** | **0.0021** |
| GM | Interaction effect between visceral adipose tissue volume (abdominal MRI) and sex | -0.0887 | 0.9293 |
| WM |  | -0.0010 | 0.9992 |
| WMH |  | -1.1683 | 0.2427 |
| Brain |  | -0.0554 | 0.9558 |
| **GM** | **Interaction effect between abdominal subcutaneous adipose (abdominal MRI)**  **tissue volume and sex** | **-4.5724** | **< 0.0001** |
| **WM** |  | **-2.6746** | **0.0075** |
| **WMH** |  | **3.5837** | **0.0003** |
| **Brain** |  | **-4.3731** | **< 0.0001** |
| **GM** | **Interaction effect between total**  **adipose tissue volume (abdominal MRI) and Sex** | **-6.7566** | **< 0.0001** |
| **WM** |  | **-3.3975** | **0.0007** |
| **WMH** |  | **4.4323** | **0.0000** |
| **Brain** |  | **-6.1603** | **< 0.0001** |
| **GM** | **Interaction effect between total lean tissue volume (abdominal MRI) and sex** | **4.2376** | **< 0.0001** |
| WM |  | 1.5545 | 0.1201 |
| WMH |  | -2.7052 | 0.0068 |
| **Brain** |  | **3.5391** | **0.0004** |
| GM | Interaction effect between  android to gynoid fat mass ratio  (DXA) and sex | 1.6942 | 0.0903 |
| WM |  | 1.2598 | 0.2078 |
| WMH |  | 0.2346 | 0.8145 |
| Brain |  | 1.7767 | 0.0757 |
| GM | Interaction effect between trunk-to-leg fat mass ratio and sex | -0.0430 | 0.9657 |
| WM |  | 0.2194 | 0.8263 |
| WMH |  | 0.3741 | 0.7084 |
| Brain |  | 0.0998 | 0.9205 |
| GM | Interaction effect between trunk-to-leg lean mass ratio and sex | -1.7677 | 0.0772 |
| WM |  | -0.8675 | 0.3857 |
| WMH |  | -0.1191 | 0.9052 |
| Brain |  | -1.5974 | 0.1102 |
| GM | Interaction effect between visceral adipose tissue mass (DXA) and sex | 0.6608 | 0.5088 |
| WM |  | 0.2701 | 0.7871 |
| WMH |  | -1.3522 | 0.1764 |
| Brain |  | 0.5637 | 0.5730 |
| **GM** | **Interaction effect between fat mass index (DXA) and sex** | **-3.9196** | **0.0001** |
| WM |  | -1.9064 | 0.0567 |
| WMH |  | 2.8847 | 0.0039 |
| **Brain** |  | **-3.5272** | **0.0004** |
| GM | Interaction effect between lean body mass index and sex | 0.5038 | 0.6145 |
| WM |  | -0.7890 | 0.4301 |
| WMH |  | 1.2422 | 0.2142 |
| Brain |  | -0.1412 | 0.8878 |

**Supplementary Table 2.** Interaction effects between various indicators of obesity and sex when predicting neuroimaging outcomes.
